# Study of the prediction of gamma passing rate in dosimetric verification of intensity-modulated radiotherapy using machine learning models based on plan complexity

**DOI:** 10.3389/fonc.2023.1094927

**Published:** 2023-07-21

**Authors:** Shizhen Bin, Ji Zhang, Luyao Shen, Junjun Zhang, Qi Wang

**Affiliations:** ^1^Radiotherapy Center, Third Xiangya Hospital of Central South University, Changsha, China; ^2^Radiotherapy Center, The Central Hospital of Shaoyang, Shaoyang, China

**Keywords:** machine learning, plan complexity, IMRT QA, dosimetric verification prediction, gamma passing rate

## Abstract

**Objective:**

To predict the gamma passing rate (GPR) in dosimetric verification of intensity-modulated radiotherapy (IMRT) using three machine learning models based on plan complexity and find the best prediction model by comparing and evaluating the prediction ability of the regression and classification models of three classical algorithms: artificial neural network (ANN), support vector machine (SVM) and random forest (RF).

**Materials and methods:**

269 clinical IMRT plans were chosen retrospectively and the GPRs of a total of 2340 fields by the 2%/2mm standard at the threshold of 10% were collected for dosimetric verification using electronic portal imaging device (EPID). Subsequently, the plan complexity feature values of each field were extracted and calculated, and a total of 6 machine learning models (classification and regression models for three algorithms) were trained to learn the relation between 21 plan complexity features and GPRs. Each model was optimized by tuning the hyperparameters and ten-fold cross validation. Finally, the GPRs predicted by the model were compared with measured values to verify the accuracy of the model, and the evaluation indicators were applied to evaluate each model to find the algorithm with the best prediction performance.

**Results:**

The RF algorithm had the optimal prediction effect on GPR, and its mean absolute error (MAE) on the test set was 1.81%, root mean squared error (RMSE) was 2.14%, and correlation coefficient (CC) was 0.72; SVM was the second and ANN was the worst. Among the classification models, the RF algorithm also had the optimal prediction performance with the highest area under the curve (AUC) value of 0.80, specificity and sensitivity of 0.80 and 0.68 respectively, followed by SVM and the worst ANN.

**Conclusion:**

All the three classic algorithms, ANN, SVM, and RF, could realize the prediction and classification of GPR. The RF model based on plan complexity had the optimal prediction performance which could save valuable time for quality control workers to improve quality control efficiency.

## 1 Introduction

Intensity-modulated radiotherapy (IMRT) is a precise radiotherapy technique that achieves a highly conformable dose distribution by adjusting the intensity distribution in the irradiation field, and it has been widely used for the precise treatment of tumors. In clinical practice, a dynamic intensity modulation method for controlling the multi-leaf collimator (MLC) to form multiple small subfields is often used. Therefore, the process of designing a dynamic IMRT plan involves continuous modulation and optimization of various parameters of each subfield, which is highly complex. To ensure the safety and accuracy of IMRT, plan quality assurance (QA) must be performed before the implementation of radiotherapy (1), among which dose verification is the most important part (2). The gamma index analysis is the most widely used evaluation method for dose verification in clinic at present (3). Studies have shown that there is a certain correlation between the complexity of the plan and the GPR (4, 5). A plan with a high complexity yields a low GPR (6). A recent review also summarized the ability of different plan complexities in 163 studies to pre-identify failed plans in patients pre-treatment QA, finally plan complexity was suggested to be used as a tool for pre-treatment QA, although it was not yet a complete replacement for pre-treatment QA (7).

Machine learning has gradually become an actively researched field in recent years owning to its efficiency and predictability. It is currently being applied in IMRT to perform tasks, such as the automatic delineation of plan target volume and organs at risk and automated treatment planning (8, 9). Machine learning has powerful capabilities for data mining, analysis and prediction. Recently, some researchers have applied the random forest algorithm to IMRT QA. For example, Sakai et al. (10) used the imaging features of the fluence map collected by EPID in IMRT QA to build a machine learning model for predicting the modeling error of MLC, and the results demonstrated that the random forest algorithm had a higher sensitivity than logistic regression model. Osman et al. (11) successfully built a proposed ANN model capable of accurately predicting the individual MLC leaf positional deviations during the dynamic IMRT delivery priori based on 14 feature parameters such as leaf planned positions, dose fraction, leaf velocity, and so on, which extracted from the planning data in the log files. Their results could be extended to actual application in the dose calculation/optimization, hence enhancing the GPR for patient-specific IMRT QA. Tomori et al. (12) used a CNN model to successfully predict GPRs for IMRT plans in prostate cancer patients based on plan dosimetry features. Lam et al. (13) applied three tree-based machine learning algorithms (AdaBoost, Random Forest, and XGBoost) to predicted gamma passing rates, and found that RF method had accurately prediction results. The results of the above studies all indicate that machine learning technology can identify failed QA plans in advance without using a linear accelerator and minimize wasted time measuring and adjusting those treatment plans which may not pass in QA, thereby improving the efficiency of radiotherapy QA and reducing QA workload.

Since planning complexity has an important influence on GPRs of plan dose verification, recent studies have begun to apply planning complexity features to predict GPRs. For example, Ono et al. (14) used plan complexity parameters to predict GPRs of volume modulated arc therapy (VMAT) plans measured by ArcCHECK *via* machine learning models, and compared the predictive performance of: regression tree analysis (RTA), multiple regression analysis (MRA) and ANN three models. Their results showed that ANN performed slightly better than RTA and MRA in terms of prediction error. While the study by Hirashima et al. (15) showed that RF had good prediction and classification performance for GPR based on plan complexity features. However, SVM machine learning model was shown to be more suitable for predicting GPR measured by MapCHECK2 diode arrays for VMAT plans based on plan complexity features (16). Thus, it can be seen that the planning complexity features selected by different studies are different, and the equipment for GPR dose verification is also different, which may affect the prediction performance of the model. Among various classical algorithms, which machine learning model is more suitable for predicting and classifying GPR of IMRT plan dose verification needs to be investigated in further study.

This study aimed to use machine learning algorithms to develop machine learning models of plan complexity features and GPR to realize the prediction of GPR in dosimetric verification of IMRT, and sought the optimal prediction model by comparing and evaluating the prediction performance of the regression and classification models of three classical algorithms: ANN, SVM and RF. The impact of each complexity feature value on the GPRs was analyzed through feature importance ranking and the number of samples required for model accuracy convergence was evaluated.

## 2 Materials and methods

### 2.1 Plan patient selection

269 clinical IMRT plans (total 2340 fields) were selected retrospectively from multiple treatment disease sites: brain (N = 34), nasopharyngeal (N = 50), lung (N = 32), breast (N = 31), esophagus (N = 31), cervical (N = 53), and rectal (N = 38). All dynamic IMRT plans were created using Varian’s Eclipse v.11.0.13 treatment plan system (Varian Medical Systems, Palo Alto, CA). Analytical anisotropic algorithm was used for dose calculation. All cases were performed using 6MV X-ray energy at a dose rate of 600MU/min, and treated with Varian’s Unique accelerator (Varian Medical Systems, Palo Alto, CA). The grid size used in calculation was 2mm.

### 2.2 Plan dosimetric verification

The EPID of the Unique accelerator provides images in digital format to detected the dose distribution with an amorphous silicon flat-panel detector. The detector has an effective detection area of 40cm×30cm^2^ with a matrix of 1024 × 768 pixels^2^, and each square pixel has a side length of 0.0392 cm. To eliminate the influence of the dose-response of the detector and the output of the accelerator on the verification result, the absolute dose calibration as well as background and general field calibrations were performed before each dose verification. The detailed steps of dose calibration were performed according to Varian’s portal dosimetry calibration operation manual, and finally 1 calibrated unit (CU) of the EPID image was equivalent to 1Gy dose. The portal dosimetry software configured in Eclipse was used to compare the actual dose distribution collected by EPID with the measurement distribution calculated using the plan system. Perpendicular field-by-field method was used to perform pre-treatment QA measurements according to AAPM Task Group 218 (17). The GPRs of 2340 beams were collected and analyzed by the 2%/2mm with a 10% maximum-dose threshold and global dose normallization which was recommended in the AAPM Task Group 119 and 218 (17, 18). When GPR was greater than 95%, the radiation therapy plan dose verification result indicated “pass”, otherwise it was “fail”. Values of measured GPRs that were used in the test and training group was discontinuously distributed, mainly in 90%~98%. 71.5% of measured GPRs values were “pass”, and the remaining was “fail”.

### 2.3 Feature parameter extraction

The plan complexity describes the degree of modulation of the complexity of the plan produced by IMRT optimization, particularly in term of the frequency and amplitude of fluctuations in the intensity distribution of a field (19), including multiple quantification methods and corresponding evaluation indicators. Based on the MLC file of each field exported from the planning system, 21 plan complexity feature values were calculated using in MATLAB R2017b programming. The specific abbreviations and definitions of all features are shown in **Table 1**. Beam irregularity (BI), beam aperture area were weighted by MU (BA), beam modulation (BM), and union of area of aperture (UAA) were defined by Du et al. (4) BI reflected the deviation of the shape of the segments relative to a circle. BM reflected the extent of a large open field being broken into multiple small segments, ranging from 0 to 1. UAA was the union area of all apertures of a beam, which was greater than or equal to the area of any individual aperture. Small aperture score (SAS) and mean asymmetry distance (MAD) were defined by Crowe et al. (20) The SAS referred to the part where small segments were used in the beam, and was calculated as the ratio of open leaf pairs where the aperture was less than a defined criteria (5, 10 and 20 mm in this study) to all open leaf pairs. The MAD was the average of the distance from the centre of every open leaf pair aperture to the central beam axis. Younge et al. (21) defined edge metric (EM), which described the ratio of MLC side-length to segments area. Modulation complexity score (MCS), leaf sequence variability (LSV), and aperture area variability (AVV) were defined by Mcniven et al. (22) MCS represented the relative variability in leaf position, segments area, and MU of each segment. LSV was the variability in segment shape. The shape of each segment was considered according to the change of leaf position between adjacent MLC leaves. AVV was the change in the area of the segments relative to the maximum field defined by all segments. The number of segments (NS), mean aperture area (MAA) and coefficient of variation of segments area (CVA) were recommended by Guo et al. (23), which were proved to be important factors affecting GPR of IMRT plan. In addition, the total number of jumps in the field (MU), Mean MU per control point (MUCP), the maximum position of the lead gate (MAXJ), the ratio of the average area of the segments to the area defined by the lead gate (MAA over the area defined by jaws, AAJA) were calculated referring to the study of Lam et al. (13) Compared to the study of Lam et al., we added three complexity feature values of NS, MAA and CVA which were the factors that had a significant impact on GPR found in actual clinical QA work. In addition, our clinical data came from the same accelerator, so we removed those features related to different equipment and different MLC modeling.

**Table 1:** Features used in machine learning prediction.

Number	Abbreviation	Interpretation
1	MU	Monitor unit
2	NS	Number of segments
3	MUCP	Mean MU per control point
4	MAA	Mean aperture area
5	SAS_5,10,20_	Mean of Small aperture score
6	MSAS_5,10,20_	Max of fraction of aperture smaller
7	CVA	Coefficient of variation of segments area
8	LSV	Leaf sequence variability
9	AVV	Aperture area variability
10	MCS	Modulation complexity score
11	MAD	Mean asymmetry distance
12	BI	Beam irregularity
13	BM	Beam modulation
14	AAJA	MAA over the area defined by jaws
15	MAXJ	Maximum of x-y jaw positions
16	EM	Edge Metric
17	UAA	Union area of aperture

### 2.4 Data preprocessing

Portal dosimetry system was used to perform a verification analysis of the dose distribution for each field to obtain GPR values. The GPR of each field and the 21 feature parameters were integrated into the original data. In essence, the GPR prediction model established by the machine learning method in this study was to use the training set with result labels to train the model, and then optimized the model to obtain the model with the highest accuracy, so as to realize the prediction of samples with unknown results. Therefore, the corresponding output variable GPR had two ways to achieve the purpose of regression and classification respectively in this study. In the regression model, the value of GPR was a continuous numerical variable; in the classification model, there were two types of GPR results ――”pass” and “fail” according to a given GPR threshold. Since the classification model was to filter out the fields that pass the dose verification, “fail” corresponds to “positive”, which was represented by “1”, and “pass” corresponds to “negative”, which was represented by “0”.

### 2.5 Model training and evaluation method

**Figure 1:**
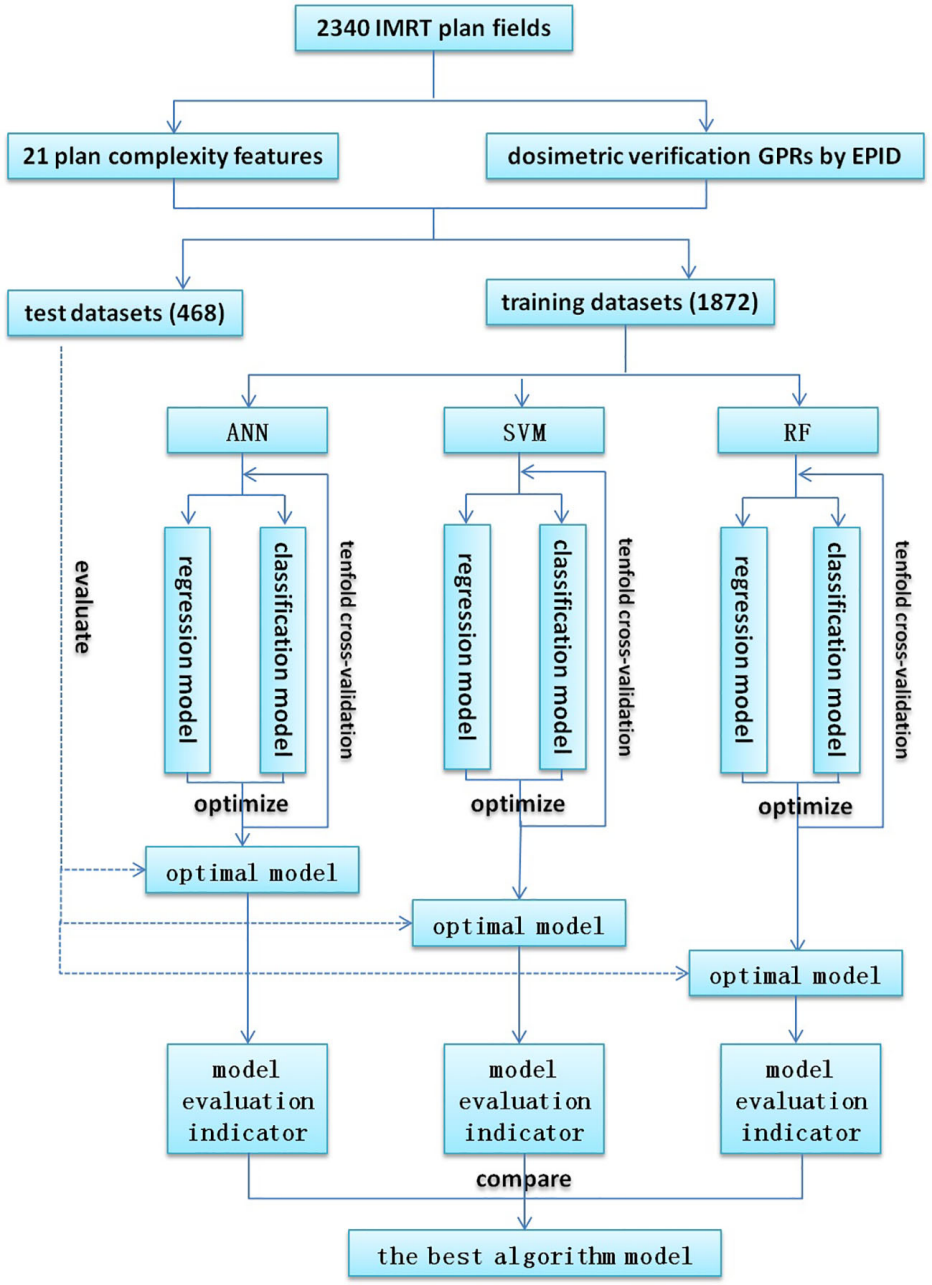
Flowchart for model training and evaluation.

The process of model training and evaluation was shown in **Figure 1**. The dataset was randomly divided into a training set (1872 fields) and a test set (468 fields) according to the ratio of 8:2, where the training set was used for model training, and the test set was used to evaluate the model performance. Because of their better prediction performance in recent studies, ANN, SVM, and RF were used to establish regression models and classification models based on the plan complexity, namely ANN-RM, SVM-RM, RF-RM and ANN-CM, SVM-CM, RF-CM. RF was an ensemble tree-based learning algorithm, which aggregated the results from a large number of decision trees to decide the final class of the test object (24, 25). SVM was a typical statistical-learning based algorithm for classification, which had good robustness and sparsity performance, and had advantages in solving small sample, nonlinear and high-dimensional classification problems (26). CNN was a network structure model composed of a large number of basic unit-neuron nodes connected with each other, as it was considered one of the most powerful ways to learn useful representations of images and other structured data (27, 28). The regression model was used to predict the value of GPR, and the classification model was used to judge whether the GPR reached the threshold. These three algorithms were provided by the nnet, e1071, and randomForest packages in the R language, respectively. The ANN algorithm provided in the nnet package can solve regression and classification problems. In actual modeling, the nnet() function was used to build a feed forward ANN model with a three-layer network structure, and the number of input nodes was equal to the number of input variables. There was only one hidden layer in the network structure, and the number of nodes needed to be set by yourself. The model was optimized by adjusting the number of nodes in the hidden layer of the model through the tune function. The SVM algorithm provided by the e1071 package could also be used to solve regression and classification models. The SVM model was constructed *via* the svm() function based on the given data structure, and was optimized by adjusting the cost and gamma parameters. Cost represented the penalty coefficient, and gamma represented the high-dimensional mapping of low-dimensional samples. The larger the gamma value, the higher the mapping dimension. The initial value defaults to cost = 1 and gamma = 0.1. After training, the optimal parameters were found by using the tune function with ten-fold cross validation within a certain range which was found by viewing the model overview. The RF algorithm provided by the randomForest package can also be used to solve regression and classification models. The RF model was constructed using the randomForest() function and optimized by adjusting the ntree and mtry hyperparameters. Ntree was the number of decision trees contained in RF, and mtry was the number of variables used in the binary tree in the node. During model training, the value of mtry with the smallest model error was first found using a for loop, and then the optimal ntree value was found using ten-fold cross validation. The feature independent variables are also randomly selected when constructing each decision tree. Each decision tree predicted the input features and obtained the predicted value. The average of these predicted values was the final prediction result. Then the out-of-bag data was used to verify the accuracy of the model’s prediction results.

For the regression model, the root mean squared error (RMSE) and mean absolute error (MAE) were used to evaluate the model. In addition, Spearman correlation analysis was performed on the predicted value and the actual GPR to obtain the correlation coefficient (CC). CC > 0.8 meant high correlation; 0.4 < CC < 0.8 meant moderate correlation; CC < 0.4 meant low correlation. For classification models, the receiver operating characteristic curve (ROC) was drawn based on the prediction results, and the area under the curve (AUC) value, sensitivity and specificity were used to evaluate the model. Finally, the optimal prediction algorithm was selected by comparison.

## 3 Results

### 3.1 Model prediction results and evaluation

#### 3.1.1 Regression model

**Figure 2:**
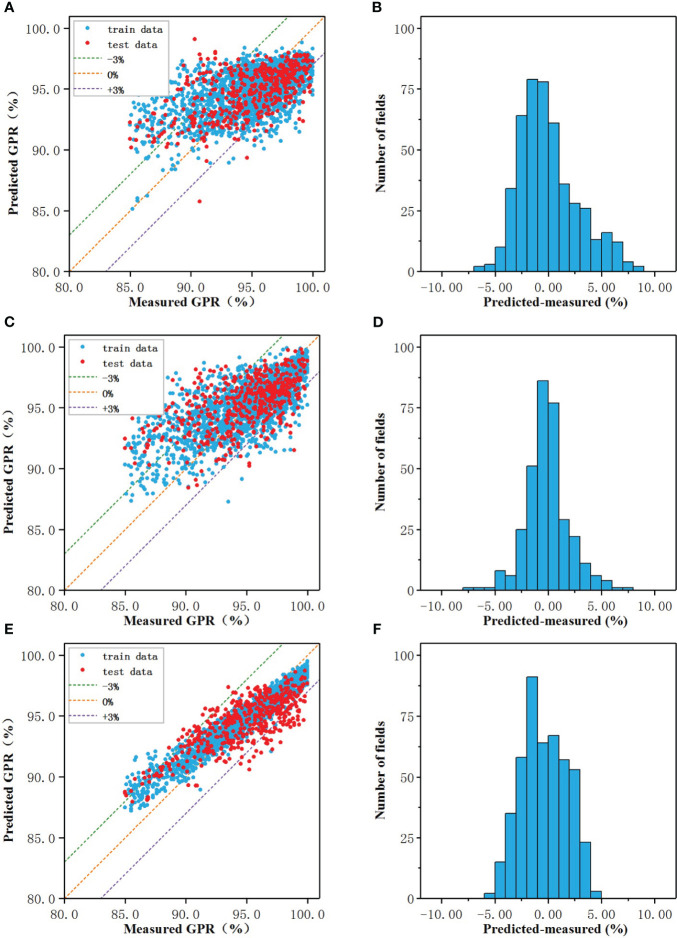
The prediction results of the regression models of three algorithms: The prediction results of the regression models of three algorithms: **(A, C, E)** Measured vs predicted GPRs, **(B, D, F)** The prediction error.

The prediction results of the regression models of three algorithms were shown in **Figures 2A, C, E**, and the performance of the models on the test set were shown in **Figures 2B, D, F**. In the scatter plot, the blue and red dots represented the correspondence between the model predicted value and the actual measured value on the training set and test set, respectively, and the three dotted lines represented the predicted value deviated from the actual value by -3% (purple), 0% (orange), +3% (green), the closer to the orange line the more accurate the predicted value.

When the number of nodes in the hidden layer was 19, the prediction result of CNN-RM was optimal. As shown in **Figure 2A**, the error of ANN-CM on the training set and the test set was partially beyond the range of ±3%, and the fitting result to the data was not ideal. For some samples with lower actual GPR, the predicted value was higher than the actual; for some samples with higher actual GPR, the predicted value was lower than the actual. Statistical analysis was carried out on the prediction error of ANN-CM on the test set. The histogram was shown in **Figure 2B**. The MAE and RMSE were 2.18% and 2.84%, respectively, and the maximum error exceeded 8%, indicating that the prediction performance of ANN-CM was poor. In addition, spearman correlation analysis was performed on the predicted GPR of ANN-CM on the test set and the actual measured value, and CC was 0.53.

When cost = 1, gamma = 0.14, the prediction result of SVM-RM was optimal, and the number of support vectors in the model was 1686. As shown in **Figure 2C**, the phenomenon that the predicted value was higher than the actual for some samples with lower actual GPR also existed, while the predicted value was lower than the actual for some samples with higher actual GPR. The error histogram of SVM-RM on the test set was shown in **Figure 2D**, and the maximum error exceeded 7%. Compared with ANN-RM, the MAE and RMSE of SVM-RM on the test set were 2.17% and 2.76%, respectively, and the prediction performance of the model had been improved to a certain extent. The CC of the test set was 0.56, which was slightly higher than ANN.

When mtry = 5, ntree = 373, the prediction result of RF-RM was optimal. As shown in **Figure 2E**, the performance of RF-CM on the training set and the test set was different. The error on the training set was almost within ±3%, but there were still samples with low actual GPR in the model on the test set, but the maximum error was within 5%. The error histogram of RF-RM on the test set was shown in **Figure 2F**. Compared with the previous two models, the prediction effect of the RF-RM had been improved, and its MAE was 1.81%, the RMSE was 2.14%, and the CC was 0.72.

As shown in **Table 2**, the RMSE, MAE, and CC of the three models were comparable. The ANN-RM had the highest RMSE and MAE on the test set, and the CC between the predicted value on the test set and the actual GPR was also the smallest. On the contrary, the RMSE and MAE of the RF regression model on the test set were the lowest, and the CC was the largest, indicating that the regression model established by the RF algorithm had the best prediction performance on GPR among the three algorithms.

**Table 2:** Performance of regression models on the test set.

Evaluation indicators	ANN-RM	SVM-RM	RF-RM
RMSE(%)	2.84	2.76	2.14
MAE(%)	2.18	2.17	1.81
CC	0.53	0.56	0.72

#### 3.1.2 Classification model

**Figure 3:**
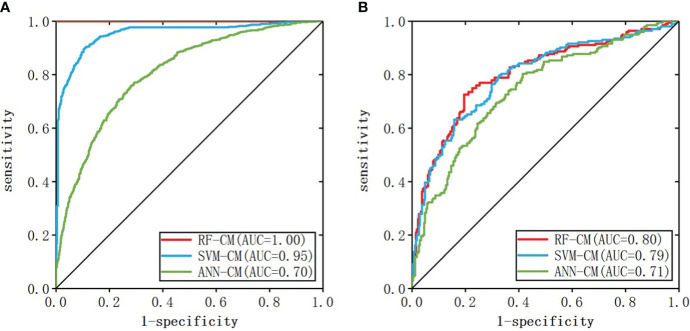
ROC curve of the classification model (**A**: training set, **B**: test set).

When the number of nodes in the hidden layer was 11 and decay = 0.1, the prediction result of ANN-CM was optimal. When cost = 8, gamma = 0.14, the prediction result of SVM-CM was optimal and the number of support vectors in the model was 1250. When mtry = 9, ntree = 256, RF-CM showed the optimal performance. The ROC curves of the three models on the training set and test set were shown in **Figure 3**, (a) was the training set, (b) was the test set, the green line represented ANN-CM, the blue line represented SVM-CM, and the red line represented RF-CM. The lines represented the RF classification model. It can be seen that the ANN-CM had the lowest ROC on the test set, and its prediction result was the worst, while the ROC curve of SVM-CM and the RF-CM on the test set were relatively close.

AUC values, specificity and sensitivity of the three models were calculated and shown in **Table 3**. The AUC value of ANN-CM was 0.71, indicating its poor predictive ability. The AUC value of SVM-CM was 0.79, and the prediction performance was greatly improved compared with ANN-CM. RF-CM had the highest AUC value of 0.80, and the prediction effect was the best among the three models. Meanwhile, compared with the other two models, RF-CM had higher specificity and sensitivity.

**Table 3:** Performance on classification models.

Evaluation indicators	ANN-CM	SVM-CM	RF-CM
AUC	0.71	0.79	0.80
specificity	0.76	0.77	0.80
sensitivity	0.53	0.66	0.68

### 3.2 Feature value importance evaluation

**Figure 4:**
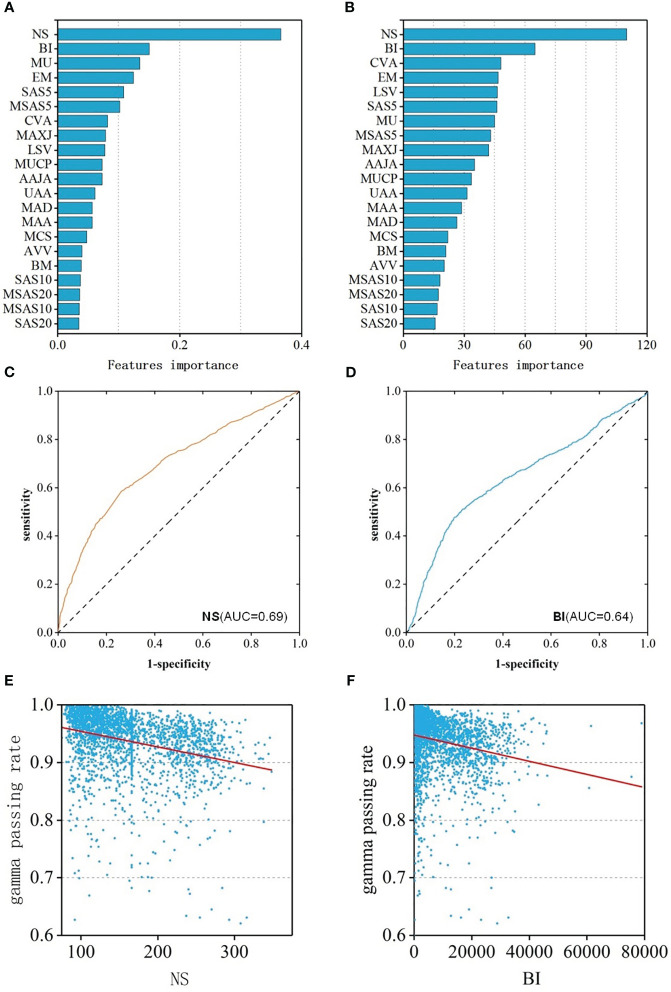
**(A)** Feature importance ranking of RF-RM; **(B)** Feature importance ranking of RF-CM; **(C)** ROC curves of NS feature; **(D)** ROC curves of BI feature; **(E)** The relationship between the NS feature and GPR; **(F)** The relationship between the BI feature and GPR.

To better reveal the impact of each feature value on the GPR, the regression and classification models of RF algorithm with the best prediction performance previous were selected to rank all features according to the importance (**Figures 4A, B**). Among them, the top three most important features were: NS、BI、MU and NS、BI、CVA MA. When the number of subfields was larger, the irregularity of the field was larger, the number of MU was more, the value of the coefficient of variation of the subfield area was larger, and lower GPR of the field.

Spearman correlation analysis was performed of the features and the GPR was performed. The results showed that the features were weakly correlated with GPR. 18 of the 21 features had a p-value of less than 0.05, indicating statistical significance. The correlation coefficient of 8 features was greater than 0.2, and the CC of NS was the largest, which was 0.40(p<0.001) with moderate correlation. The CC of BI was -0.33 (p<0.001), ranking second, showing a weak correlation. In addition, to assess the ability to identify plans that “fail” for dose validation based on these two features alone, ROC analysis was performed on these two features separately, and ROC curves were drawn in **Figures 4C, D**. The AUC values of NS and BI were 0.69 and 0.64, respectively. The two features were consistent with the results of the feature importance ranking, and the relationship between feature and GPR were shown in **Figures 4E, F**.

### 3.3 Impact of the number of samples

**Figure 5:**
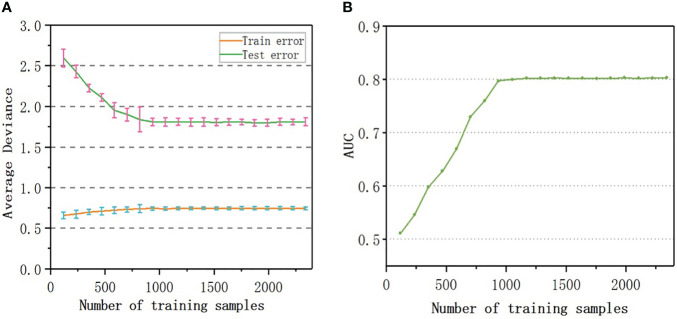
Learning curve: **(A)** Testing and Training average deviance versus number of samples used to build RF-RM model; **(B)** The relationship between the AUC value and the number of samples used to train RF-CM.

To evaluate the number of samples required for the convergence of the accuracy of the model, samples equivalent to 5%, 10%, 15%,…, 100% of the original sample size were randomly selected as training data to build 20 sets of models, the prediction error was calculated, and the learning curves were drawn. As shown in **Figure 5A**, the number of samples rarely affected the training error; the test error decreased with an increase in the sample size. When the sample number reached 800, the predicted error of RF-RM was close to the minimum (1.81%) and tended to be stable. When the sample number exceeded 800, the accuracy of the prediction reached the upper limit, and the performance of the model could not be further improved.

The AUC value of RF-CM increased with the increase of the number of samples (**Figure 5B**). When the number of training samples exceeded 900, the AUC value reached the upper limit of 0.80, and the performance of the model would not be further improved.

## 4 Discussion

In this study, 21 planning complexity features of 2340 fields of 269 patients with different diseases were calculated and extracted as input data, and the regression and classification models were constructed by using ANN, SVM, and RF algorithms. All the three algorithms realized the prediction of GPR in dosimetric verification of IMRT plan. Among the three regression models, RF-RM had the optimal prediction performance on GPR and most recommended, the RMSE and MAE on the test set were the lowest, and CC was the largest. RF-RM achieved relatively accurate prediction of GPR with a maximum error of less than 5%. This was consistent with the results demonstrated by Lam et al. (13), which indicated that the GPR of IMRT QA measured by portal dosimetry could be accurately predicted using a tree-based ensemble learning model, and showed that RF method had the optimal prediction results. However, the prediction results of SVM-RM and ANN-RM were not very ideal, the maximum error exceeded 7%, and CC was as low as 0.53~0.56, which was basically the same as the research results (CC = 0.50~0.58) of Ono et al. (14). In addition, all three models over predicted samples with lower actual GPR and under predicted samples with higher actual GPR, which might be caused by data imbalance (29). There were relatively few GPR samples greater than 98% and less than 90% in actual clinically measured datasets. Therefore, the under sampling approach which removes a fraction of the majority samples and the over sampling approach which duplicates the minority samples can be used to re-balance the data distribution for improvement in subsequent research (30).

Among the classification models, the prediction performance of ANN-CM was poor, while both RF-CM and SVM-CM could classify GPR well, and RF-CM showed higher sensitivity and specificity, and had excellent classification performance. This was also confirmed in the research results of Li et al. (31): RF model with 100% sensitivity was preferred for the classification of QA results. The AUC value of RF-CM reached 0.80, which was better than the classification result of the complexity-based model of Hirashima et al. (15) (AUC = 0.73).

To sum up, compared with ANN and SVM, the regression model and the classification model constructed by RF had better prediction performance on GPR of IMRT plan dose verification result. A typical advantage of random forests as an ensemble algorithm was the ability to aggregate resulting from a large number of decision trees that utilized various combinations of features using a random bagging scheme to give each feature a chance to be considered in the final model (24, 25). To a certain extent, RF model achieved relatively accurate prediction of GPR, but there were still difficulties in practical clinical practice. The patient treatment must be carried out after the plan dose verification QA was passed, so the prediction and recognition ability of the “fail” plan that could not pass the dose verification should better be close to 100%. However, the current model cannot achieve, it could only help reduce the workload of dose verification QA instead of completely replacing. In addition, this model was built based on planning complexity features. In actual clinical practice, different linacs and different verification devices might affect the verification results. Therefore, this model can only provide a modularization reference of planning complexity features for the development of an automated QA dose verification prediction tool which can be applied to clinical practice.

In addition, based on the high complexity of the dynamic IMRT plan, the plan complexity was quantified by combining different mathematical formulas used in various studies and 21 characteristic parameters were extracted. The top factors that had the greatest impact on the GPRs were determined through the feature importance ranking analyzed by RF algorithm in this study. Among them, NS had the greatest impact on GPR and the Spearman CC was 0.40 (p < 0.001), which was negatively correlated. Jubbier et al. (32) and Chi et al. (33) found that a large number of subfields led to a greater deviation and lower GPR, which was consistent with our findings. Therefore, when the IMRT plan was designed, the GPR of dose verification could be improved by controlling the number of subfields. In addition, BI also had a significant impact on the GPR, which depended on the shape of the subfield and quantifies the narrowness of the subfield (4). The results showed that a narrow subfield led to low GPR when designing IMRT plans with a sliding window dynamic intensity modulation technology. With the continuous improvement of clinical requirements for dose distribution, the subfield of dynamic IMRT was gradually narrowing, and a narrow the subfield led to a greater deviation. Therefore, at the design plan stage, it might be necessary to make a trade-off between the dose distribution and dose accuracy. It can also provide direction for improving the plan design optimization. If the predicted GPR does not meet the clinical requirements, the complexity of the plan can be reduced by adjusting the features that have a high correlation with the GPR. Thus, the consistency between the planned dose distribution, and the actual dose distribution can be improved.

Furthermore, our increased complexity feature CVA was also shown to affect GPR, consistent with findings in real clinical QA work. Studies had shown that a large CVA, corresponds to a greater degree of dispersion of the subfield area, as well as, higher complexity and lower GPR of the field (23). Several other factors affect the GPR of plan verification. It remains to be investigated whether adding other feature parameters can improve the prediction accuracy of the model.

Correlations between GPR and different complexity features vary. For example, the research results of Crowe et al. (20) showed that the QA results of the IMRT fields were significantly related to MCS, SAS, MAD. The study of Gödtstedt et al. (5) showed that the QA results of the IMRT fields had a strong correlation with EM and MU, a moderate correlation with MAA, and a weak correlation with MCS and BI. Our results also showed that the NS complexity feature was moderately correlated with GPR. For other QA systems, although some of the complexity indexes were not related to GPR in previous studies (22, 34), most of the plan complexity metrics were moderately correlated or weakly correlated. This might be due to the increased complexity of other QA systems in the QA operation process and the greater differences of human operation between different centers.

The number of samples directly affects the prediction accuracy of the model. In the RF-RM constructed by Lam et al. (13), the accuracy of the model on the test set could reach a high level and became stable when the number of training samples exceeded 1000 while in this study, the machine learning model of RF-RM and RF-CM used required 800 fields and 900 fields to achieve the best prediction accuracy. The reason why this result was slightly better than that of Lam et al. might be due to the analysis of verification data from only one accelerator in this study, which also showed that if the dataset was uniform, small sample size was required to build a model with high accuracy.

This study was a single-center study on single linac types of equipment without multi-center validation, which was its limitation. Whether this model had the same results in different treatment planning systems and accelerators in different centers needs further study. Although the conventional quality assurance of the accelerator, including mechanical, dose and EPID imaging equipment, had been implemented according to AAPM TG142 report (35), the uncertainty of portal dosimetry delivery was not considered which could be improved in the future clinical dose verification and research as follow: a previous plan was selected randomly for the second dose verification before each verification, and then compared the GPRs results with the first delivery (13). Studies had shown that expanding the category and number of feature values could improve the prediction accuracy of the model (36, 37). Therefore, if the model could integrate the plan dosimetry or imageomics and other features, while increasing the number of samples, then the maximum error of prediction could be further reduced and the prediction accuracy can be improved. In addition, the parameter adjustment of the model in the training process was performed manually. If the optimal parameters generated based on an optimization theory could be automatically completed, the model would be easier to be applied in different centers.

## 5 Conclusion

In summary, three classic algorithms, ANN, SVM, and RF, were used in this study to establish machine learning models, which realized the prediction and classification of GPR. The results of the comparison and evaluation of the models showed that RF model based on plan complexity had the optimal prediction performance which could save valuable time for quality control workers to improve quality control efficiency.

## Data availability statement

The raw data supporting the conclusions of this article will be made available by the authors, without undue reservation.

## Ethics statement

The studies involving human participants were reviewed and approved by 2019-S196. The patients/participants provided their written informed consent to participate in this study.

## Author contributions

Conception and design: SB, QW. Acquisition of data: SB, LS, JJZ. Analysis of data: JZ, LS. Writing the first draft of the manuscript: SB, QW and JJZ. All authors contributed to the article and approved the submitted version.
